# Characteristics of Patients in SPCG-15—A Randomized Trial Comparing Radical Prostatectomy with Primary Radiotherapy plus Androgen Deprivation Therapy in Men with Locally Advanced Prostate Cancer

**DOI:** 10.1016/j.euros.2022.04.013

**Published:** 2022-05-26

**Authors:** Magdalena Gongora, Johan Stranne, Eva Johansson, Matteo Bottai, Camilla Thellenberg Karlsson, Klaus Brasso, Steinbjørn Hansen, Henrik Jakobsen, Fredrik Jäderling, Henriette Lindberg, Wolfgang Lilleby, Peter Meidahl Petersen, Tuomas Mirtti, Mats Olsson, Antti Rannikko, Martin Andreas Røder, Per Henrik Vincent, Olof Akre

**Affiliations:** a Department of Molecular Medicine and Surgery, Karolinska Institutet, Stockholm, Sweden; b Department of Pelvic Cancer, Karolinska University Hospital, Stockholm, Sweden; c Department of Urology, Sahlgrenska University Hospital, Region Västra Götaland, Gothenburg, Sweden; d Department of Urology, Institute of Clinical Sciences, Sahlgrenska Academy, University of Gothenburg, Gothenburg, Sweden; e Department of Surgical Sciences, Uppsala University, Uppsala, Sweden; f Division of Biostatistics, Institute of Environmental Medicine, Karolinska Institutet, Stockholm, Sweden; g Department of Radiation Sciences, Oncology, Umeå University, Umeå, Sweden; h Department of Urology, Center for Cancer and Organ Diseases, Copenhagen Prostate Cancer Center, Copenhagen University Hospital – Rigshospitalet, Copenhagen, Denmark; i Department of Clinical Medicine, University of Copenhagen, Copenhagen, Denmark; j Department of Clinical Research, University of Southern Denmark, Odense, Denmark; k Department of Oncology, Odense University Hospital, Odense, Denmark; l Department of Urology, Herlev Hospital, University of Copenhagen, Copenhagen, Denmark; m Department of Radiology, Capio S:t Görans Hospital, Stockholm, Sweden; n Department of Oncology, Copenhagen University Hospital − Herlev and Gentofte, Herlev, Denmark; o Radiumhospital, Oslo University Hospital, Oslo, Norway; p Department of Oncology, Copenhagen University Hospital, Rigshospitalet, Denmark; q Department of Pathology, University of Helsinki and Helsinki University Hospital, Helsinki, Finland; r Research Program in Systems Oncology, Faculty of Medicine, University of Helsinki, Helsinki, Finland; s iCAN-Digital Precision Cancer Medicine Flagship, Helsinki, Finland; t Department of Clinical Science, Intervention and Technology, Karolinska Institutet, Stockholm, Sweden; u Department of Urology, Faculty of Medicine and Helsinki University Hospital, University of Helsinki, Helsinki, Finland

**Keywords:** Prostatic neoplasms, Locally advanced prostate cancer, Radical prostatectomy, Radiotherapy, Androgen deprivation, Curative treatment, Quality of life, Randomized

## Abstract

**Background:**

There is no high-grade evidence for surgery as primary treatment for locally advanced prostate cancer. The SPCG-15 study is the first randomized trial comparing surgical treatment with radiotherapy.

**Objective:**

To describe the baseline characteristics of the first 600 randomized men in the SPCG-15 study. The study will compare mortality and functional outcomes.

**Design, setting, and participants:**

This study is a Scandinavian prospective, open, multicenter phase III randomized clinical trial aiming to randomize 1200 men.

**Intervention:**

Radical prostatectomy with or without consecutive radiotherapy (experimental) and radiotherapy with neoadjuvant androgen deprivation therapy (standard of care).

**Outcome measurements and statistical analysis:**

Cause-specific survival, metastasis-free survival, overall survival, and patient-reported bowel function, sexual health, and lower urinary tract symptoms were measured.

**Results and limitations:**

The distribution of characteristics was similar in the two study arms. The median age was 67 yr (range 45–75 yr). Among the operated men, 36% had pT3a stage of disease and 39% had pT3b stage. *International Society of Urological Pathology* grades 2, 3, 4, and 5 were prevalent in 21%, 35%, 7%, and 27%, respectively. Half of the men (51%) in the surgery arm had no positive lymph nodes. The main limitation is the pragmatic design comparing the best available practice at each study site leading to heterogeneity of treatment regimens within the study arms.

**Conclusions:**

We have proved that randomization between surgery and radiotherapy for locally advanced prostate cancer is feasible. The characteristics of the study population demonstrate a high prevalence of advanced disease, well-balanced comparison groups, and a demography mirroring the Scandinavian population of men with prostate cancer at large.

**Patient summary:**

This study, which has recruited >600 men, compares radiotherapy with surgery for prostate cancer, and an analysis at the time of randomization indicates that the study will be informative and generalizable to most men with locally advanced but not metastasized prostate cancer.

## Take Home Message

We present the first randomized controlled trial comparing prostatectomy with radiotherapy plus androgen deprivation therapy among men with locally advanced prostate cancer. We have proved the feasibility of the randomized approach and present baseline characteristics of the first 600 randomized men.

## 1 Introduction

Prostate cancer mortality has declined significantly over the past 15 yr, mainly due to earlier detection and treatment through opportunistic testing with prostate-specific antigen (PSA) [1]. Around 3.5–5% of men diagnosed with prostate cancer are diagnosed with a locally advanced, defined as stage T3 or T4, nonmetastatic cancer [2], [3]. Locally advanced prostate cancer is associated with a high risk of progression and subsequent prostate cancer mortality [4].

In contrast to other major cancer forms, there has been a long-standing lack of multimodal cancer treatments for locally advanced prostate cancer. For a long time, there was no evidence-based curative treatment, and many men with locally advanced nonmetastatic prostate cancer were treated conservatively with androgen deprivation therapy alone [4]. In 2009, the SPCG-7 trial reported a clear benefit of radiotherapy with androgen deprivation therapy in men with mainly locally advanced prostate cancer, compared with androgen deprivation therapy alone, and the findings have since been corroborated further by two additional randomized trials [5], [6], [7].

While there is evidence from randomized trials for survival benefit of surgery in localized prostate cancer, and data from observational cohorts support radical prostatectomy as treatment for locally advanced disease, there is yet no randomized controlled trial comparing primary surgery with primary radiotherapy for men with mainly locally advanced prostate cancer [7], [8], [9], [10], [11], [12], [13], [14], [15], [16], [17], [18].

SPCG-15 is a Scandinavian multicenter, randomized, open phase III trial in which patients are randomized 1:1 either to primary radiotherapy including androgen deprivation or to primary radical prostatectomy with optional postoperative radiotherapy [19].

Here, we present the baseline characteristics and pathology data from the prostatectomy specimen of the first 600 patients. Our aim is to describe the characteristics of the study cohort to assess future generalizability.

## 2 Patients and methods

SPCG-15 is a prospective, multicenter, open, randomized phase III trial including patients with locally advanced, stage T3 or T4 (according to either clinical examination or radiology; defined on magnetic resonance imaging [MRI] as a risk of extraprostatic extension of at least 4/5 on a Likert’s scale [20]), N0 stage (defined in accordance to the RECIST guidelines [<1.5 cm long axis]) [21], nonmetastatic (confirmed by bone scan, computed tomography, or MRI of axial skeleton, at a minimum of pelvis and lumbar vertebral column [22]) prostate cancer randomized either to radiotherapy with neoadjuvant as well as adjuvant (hereafter, for brevity, referred to as neoadjuvant) androgen deprivation (standard arm) or to radical prostatectomy followed by salvage radiation or endocrine therapy if deemed indicated (experimental arm).

The randomization is done in blocks to ensure an even distribution within study sites. The study protocol has been described in more detail previously (for full inclusion and exclusion criteria, please see the article describing the SPCG-15 trial by Stranne et al. [19]). The total number of randomized patients aimed for inclusion in the study is 1200. The primary aim is to investigate whether radical prostatectomy with postoperative radiotherapy if needed improves prostate cancer–specific survival in comparison with primary radiotherapy plus androgen deprivation. Secondary aims are to compare metastasis-free and overall survival, quality of life, functional outcomes, and healthcare consumption in the two treatment arms.

Before randomization, patients fill out a study-specific quality-of-life questionnaire. The form was created using a “one concept, one question” method described in detail before by Steineck et al. [23], and adjusted after interviews with prostate cancer patients and tested for face validity.

The quality-of-life questionnaire comprises 118 questions concerning patient characteristics, demography, experience, psychological symptoms (anxiety and depression), sense of well-being, and quality of life, as well as physical symptoms (including urinary, bowel, and sexual functions), pain, and, in addition, symptoms regarding hormonal therapy, if applicable. This form is filled in at baseline as well as 1, 2, 5, 10, 15, and 20 yr after randomization. The questions investigate the quality, frequency, and intensity of each symptom and include a bother score. The quality-of-life and psychological parameters are measured on a visual digital scale from 1 to 7, where 1 and 2 are considered low intensity; 3, 4, and 5 moderate intensity, and 6 and 7 high intensity.

Follow-up, including laboratory tests and clinical examination, is documented using web-based case report forms including all data pertaining to primary and secondary endpoints. The case report forms are filled out by study nurses and investigators at each site.

The first patient was randomized in October 2014, and, as of May 23rd 2022 856 patients have been randomized. In the present analysis, we have chosen to describe the first 600 patients to avoid missing data due to latency in reporting.

The study was financed by grants from the Swedish Research Council (dnr 2017-00546), the Nordic Cancer Union, and the Swedish state under the agreement between the Swedish government and the county councils (the ALF agreement; grant number FoUI-953889).

Patient data were collected with written informed consent. The study was approved by the national ethical review authorities in Sweden, Norway, Finland, and Denmark.

## 3 Results

Baseline demographic and social characteristics are presented in Table 1. The mean age at randomization was 67 yr (range 45–75 yr). Of the cohort, 66% are either overweight or obese, and a majority (81%) are nonsmokers; 84% exercise at least 1–3 h/wk.

**Table 1:** Socioeconomic characteristics at baseline

Socioeconomics	Prostatectomy (%)	Radiotherapy (%)	Total (%)
Number of patients	301 (100)	299 (100)	600 (100)
Inclusion country			
Sweden	103 (34)	97 (32)	200 (33)
Norway	44 (15)	49 (16)	93 (16)
Denmark	115 (38)	115 (38)	230 (38)
Finland	39 (13)	38 (13)	77 (13)
Missing	0 (0)	0 (0)	0 (0)
Age (at inclusion)			
Median (yr)	68	68	68
<56 yr	7 (2)	15(5)	21 (4)
56–60 yr	28 (9)	30 (10)	58 (10)
61–65 yr	62 (21)	44 (15)	106 (18)
66–70 yr	105 (35)	116 (39)	221 (37)
71–75 yr	95 (32)	90 (30)	185 (31)
Missing	4 (1)	5 (2)	9 (2)
Marital status			
Married or cohabitating	228 (76)	232 (78)	460 (77)
Living alone without partner	27 (9)	26 (9)	53 (9)
Living alone with partner, that is, living apart	21 (7)	20 (7)	41 (7)
Widower	7 (2)	5 (2)	12 (2)
Missing	18 (6)	16 (5)	34 (6)
BMI			
<18.5	1 (0)	0 (0)	1 (0)
18.5–25	80 (27)	90 (30)	170 (28)
>25–30	142 (47)	139 (46)	281 (47)
>30	61 (20)	52 (17)	113 (19)
Missing	17 (6)	18 (6)	35 (6)
Occupation			
Working	78 (26)	84 (28)	162 (27)
Retired	194 (64)	187 (63)	381 (64)
Long-term ill	5 (2)	2 (1)	7 (1)
Retired due to illness	5 (2)	7 (2)	12 (2)
Unemployed	2 (1)	2 (1)	4 (1)
Missing	17 (6)	17 (6)	34 (6)
Education			
Lower secondary school or equivalent	70 (23)	84 (28)	154 (26)
Upper secondary school or equivalent	128 (43)	111 (37)	239 (40)
University or collage	87 (29)	86 (29)	173 (29)
Missing	16 (5)	18 (6)	34 (6)
Physical activity			
<1 h/wk	34 (11)	32 (11)	66 (11)
1–3 h/wk	89 (30)	97 (32)	186 (31)
>3 h/wk	162 (54)	154 (52)	316 (53)
Missing	16 (5)	16 (5)	32 (5)
Smoking habits			
Nonsmoker	249 (83)	235 (79)	484 (81)
Smoker	34 (11)	44 (15)	78 (13)
Smoker (unknown amount)	2 (5)	5 (11)	7 (9)
Smoker (1–10 cigarettes/d)	15 (44)	24 (55)	39 (50)
Smoker (≥11 cigarettes/d)	17 (50)	15 (34)	32 (41)
Missing	18 (6)	20 (7)	38 (6)
Alcohol consumption			
Median alcohol consumption	2–3 times/wk	2–4 times/mo	2–3 times/wk

BMI = body mass index.

Table 2 presents diagnostic data for both study arms. For the men in the surgery arm, pathological characteristics of the specimen are presented in Table 3. In general, the distribution of clinical characteristics in the two treatment arms were similar. The median and mean PSA for the whole study population were 12.0 and 18.9 µg/l, respectively. Of the patients, 82% had palpable clinical T3 disease. The remaining patients have been included exclusively based on the signs of extracapsular tumor on MRI. In the postoperative pathology review of the surgical specimen, 16% had intracapsular disease (pT2) only. A total of 75% had pT3 disease postoperatively, and of them a slight majority were classified to have pT3b (39%). Of the patients, 26% had a biopsy International Society of Urological Pathology (ISUP) grade of 3 and 19% had an ISUP grade of 4. Postoperative pathology report showed that 35% ISUP grade 3 patients, 7% ISUP grade 4 patients, and 80% of the patients had no or fewer than four positive lymph nodes.

**Table 2:** Clinical characteristics at baseline

Clinical baseline data	Prostatectomy (%)	Radiotherapy (%)	Total (%)
Number of patients	301 (100)	299 (100)	600 (100)
Previous active surveillance			
Yes	15 (5)	15 (5)	30 (5)
No	268 (89)	263 (88)	531 (88)
Missing	18 (6)	21 (7)	39 (7)
PSA at inclusion (µg/l)			
Median	13.0	11.0	12.0
Interquartile range (25p–75p)	7.5–26.0	7.3–23.0	7.5–24.8
% PSA >20	34	28	31
Missing	0 (0)	0 (0)	0 (0)
Clinical T stage (DRE)			
T1	19 (6)	20 (7)	39 (7)
T2	33 (11)	34 (11)	67 (11)
T3	248 (82)	244 (82)	492 (82)
T4	1 (0)	0 (0)	1 (0)
Missing	0 (0)	1 (0)	0 (0)
Imaging T stage (MRI)			
No. of patients undergoing MRI	179	182	361
T1	3 (2^a^)	1 (1^a^)	4 (1^a^)
T2	6 (3) a	4 (2^a^)	10 (3^a^)
T3	163 (91^a^)	167 (92^a^)	330 (91^a^)
T4	7 (4^a^)	10 (5^a^)	17 (5^a^)
Missing	0 (0^a^)	0 (0^a^)	0 (0^a^)
Prostate volume (cc)			
Median	38.5	36.0	37.0
IQ range (25p–75p)	30.0–50.5	30.0–45.0	30.0–48.9
% >50 cc	25	16	20,0
Missing	17 (6)	20 (7)	37 (6)
ISUP grade			
Grade group 1	0 (0)	0 (0)	0 (0)
Grade group 2	78 (26)	76 (25)	154 (26)
Grade group 3	87 (29)	80 (27)	168 (28)
Grade group 4	49 (16)	62 (21)	111 (19)
Grade group 5	87 (29)	79 (26)	166 (28)
Missing	0 (0)	2 (1)	2 (0)
Biopsies			
No of cores^b^			
2–5	17 (6)	27 (9)	44 (7)
6–9	29 (10)	27 (9)	56 (9)
10–14	238 (79)	235 (79)	473 (79)
≥15	6 (2)	2 (1)	8 (1)
Missing	11 (4)	8 (3)	19 (3)
Total biopsy length (mm)			
<50	17 (6)	30 (10)	47 (8)
50–99	29 (10)	32 (11)	61 (10)
100–199	208 (69)	189 (63)	397 (66)
≥200	20 (7)	26 (9)	46 (8)
Missing	27 (9)	22 (7)	49 (8)
Number of cores with cancer			
1–4 biopsies	60 (20)	82 (27)	142 (24)
5–9 biopsies	147 (49)	144 (48)	291 (49)
≥10 biopsies	81 (27)	63 (21)	144 (24)
Missing	13 (4)	10 (3)	23 (4)
Total cancer length (mm)			
<20	44 (15)	53 (18)	97 (16)
20–49	101 (34)	105 (35)	206 (34)
50–99	103 (34)	93 (31)	196 (33)
100–149	29 (10)	28 (9)	57 (10)
>150	7 (2)	6 (2)	13 (2)
Missing	17 (6)	14 (5)	31 (5)
Charlson comorbidity index^c^			
0–2	29 (10)	33 (11)	62 (10)
3–4	228 (76)	220 (74)	448 (75)
>4	29 (10)	33 (11)	28 (5)
Missing	15 (5)	13 (4)	28 (5)

CCI = Charlson comorbidity index; DRE = digital rectal examination; IQ = interquartile; ISUP = International Society of Urological Pathology; MRI = magnetic resonance imaging; PSA = prostate-specific antigen.

a % out of patients undergoing MRI.

b Low number of biopsies mainly due to fusion or targeted biopsies.

c No points for prostate cancer in CCI tumor.

**Table 3:** – Pathology data from the surgical specimen

Postoperative data	Prostatectomy (%)
Number of patients	301 (100)
T stage (pT)	
T2	47 (16)
T3a	107 (36)
T3b	118 (39)
T4	2 (1)
Missing	27 (9)
ISUP grade	
Grade group 1	2 (1)
Grade group 2	62 (21)
Grade group 3	106 (35)
Grade group 4	22 (7)
Grade group 5	82 (27)
Missing	27 (9)
Lymph nodes^a^	
Lymph node status	
Positive	111 (37)
Negative	153 (51)
Missing	37 (12)
No. of positive lymph nodes	
0 lymph nodes	153 (51)
1–3 lymph nodes	88 (29)
>3 lymph nodes	24 (8)
Missing	36 (12)

ISUP = International Society of Urological Pathology.

a Seventy-nine patients had both pT3b and positive lymph node status.

Of the men, 95% completed the baseline quality-of-life questionnaire. Table 4, Table 5 show the distribution of key characteristics from the quality-of-life questionnaire at baseline. Of all men in the trial, 43% and 48% reported moderate to high depression and anxiety, respectively.

**Table 4:** QoL data at baseline

Quality of life data	Prostatectomy (%)	Radiotherapy (%)	Total (%)
Number of patients	301 (100)	299 (100)	600 (100)
Baseline quality of life			
Low intensity	13 (4)	4 (1)	17 (3)
Moderate intensity	133 (48)	143 (48)	276 (46)
High intensity	138 (46)	136 (45)	274 (46)
Missing	17 (6)	16 (5)	33 (6)
Meaningfulness			
Low intensity	5 (2)	2 (1)	7 (1)
Moderate intensity	79 (26)	79 (26)	158 (26)
High intensity	199 (66)	202 (68)	401 (67)
Missing	18 (6)	16 (5)	34 (6)
Mental well-being			
Low intensity	18 (6)	11 (4)	29 (5)
Moderate intensity	136 (45)	148 (49)	284 (47)
High intensity	128 (43)	125 (42)	253 (42)
Missing	19 (6)	15 (5)	34 (6)
Depression			
Low intensity	155 (51)	155 (52)	310 (52)
Moderate intensity	108 (36)	111 (37)	219 (37)
High intensity	20 (7)	17 (6)	37 (6)
Missing	18 (6)	16 (5)	34 (6)
Anxiety			
Low intensity	133 (44)	143 (48)	276 (46)
Moderate intensity	122 (41)	108 (36)	230 (38)
High intensity	27 (9)	32 (11)	59 (10)
Missing	19 (6)	16 (5)	35 (6)

QoL = quality of life.

Patients answer question on a scale from 1 to 7, with 1–2 indicating low intensity, 3–5 moderate intensity, and 6–7 high intensity.

**Table 5:** Description of sex life and symptoms from the lower urinary tract and bowel at baseline

LUTS, bowel, sex	Prostatectomy (%)	Radiotherapy (%)	Total (%)
Number of patients	301 (100)	299 (100)	600 (100)
Urinary tract symptoms			
Weak urine stream			
Never	53 (18)	75 (25)	128 (21)
In less than half of the times	105 (35)	105 (35)	210 (35)
In more than half of the times	75 (25)	60 (20)	135 (23)
Always	50 (17)	42 (14)	92 (15)
Missing	19 (6)	17 (6)	35 (6)
Nocturia			
Never	46 (15)	44 (15)	90 (15)
1 per night	118 (39)	118 (39)	236 (39)
2 per night	75 (25)	63 (21)	138 (23)
3 per night	26 (9)	34 (11)	60 (10)
4 per night	12 (4)	16 (5)	28 (5)
≥5 per night	3 (1)	6 (2)	9 (2)
Missing	21 (7)	18 (6)	39 (7)
Urinary leakage			
Never	233 (77)	243 (81)	476 (79)
Once per month or more	25 (8)	20 (7)	45 (8)
At least once per week	8 (3)	12 (4)	20 (3)
At least 3 times per week	7 (2)	3 (1)	10 (2)
At least once per day	7 (2)	3 (1)	10 (2)
At least 2 times per day	2 (1)	1 (0)	3 (1)
Always	0 (0)	0 (0)	0 (0)
Missing	19 (6)	17 (6)	36 (6)
Urinary catheter			
Yes	6 (2)	6 (2)	12 (2)
No	271 (90)	272 (91)	543 (91)
Missing	24 (8)	21 (7)	45 (8)
Overall bother			
No bother	154 (51)	148 (49)	302 (50)
A little bother	83 (28)	71 (24)	154 (26)
Moderate bother	36 (12)	40 (13)	76 (13)
Considerable bother	9 (3)	19 (6)	28 (5)
Missing	19 (6)	21 (7)	40 (7)
Bowel symptoms			
Constipation			
Never	216 (72)	225 (75)	441 (74)
At least once per month	45 (15)	40 (13)	85 (14)
At least once per week	17 (6)	14 (5)	31 (5)
At least 3 times per week	2 (1)	3 (1)	5 (1)
At least once per day	2 (1)	0 (0)	2 (0)
At least 2 times per day	0 (0)	0 (0)	0 (0)
Always (almost)	2 (1)	0 (0)	2 (0)
Missing	17 (6)	17 (6)	34 (6)
Loose stool			
Never	153 (51)	154 (52)	307 (51)
At least once per month	59 (20)	75 (25)	134 (22)
At least once per week	40 (13)	34 (11)	74 (12)
At least 3 times per week	19 (6)	11 (4)	30 (5)
At least once per day	3 (1)	3 (1)	6 (1)
At least 2 times per day	2 (1)	1 (0)	3 (1)
Always (almost)	8 (3)	3 (1)	11 (2)
Missing	17 (6)	18 (6)	35 (6)
Sex life			
Ability to achieve erection			
Nonexistent	45 (15)	48 (16)	93 (16)
Inadequate for any kind of sexual activity	51 (17)	45 (15)	96 (16)
Sufficient for masturbation	80 (27)	73 (24)	153 (26)
Sufficient for intercourse	103 (34)	114 (38)	217 (36)
Missing	22 (7)	19 (6)	41 (7)
Ability to achieve orgasm			
Very poor to nonexistent	44 (15)	41 (14)	85 (14)
Poor	50 (17)	49 (16)	99 (17)
Moderate	75 (25)	79 (26)	154 (26)
Good	68 (23)	57 (19)	125 (21)
Very good	38 (13)	47 (16)	85 (14)
Missing	26 (9)	26 (9)	52 (9)
Active sex life with partner			
Yes	168 (56)	176 (59)	344 (57)
No	111 (37)	102 (34)	213 (36)
Missing	22 (7)	21 (7)	43 (7)

LUTS = lower urinary tract symptoms.

**Fig. 1:**
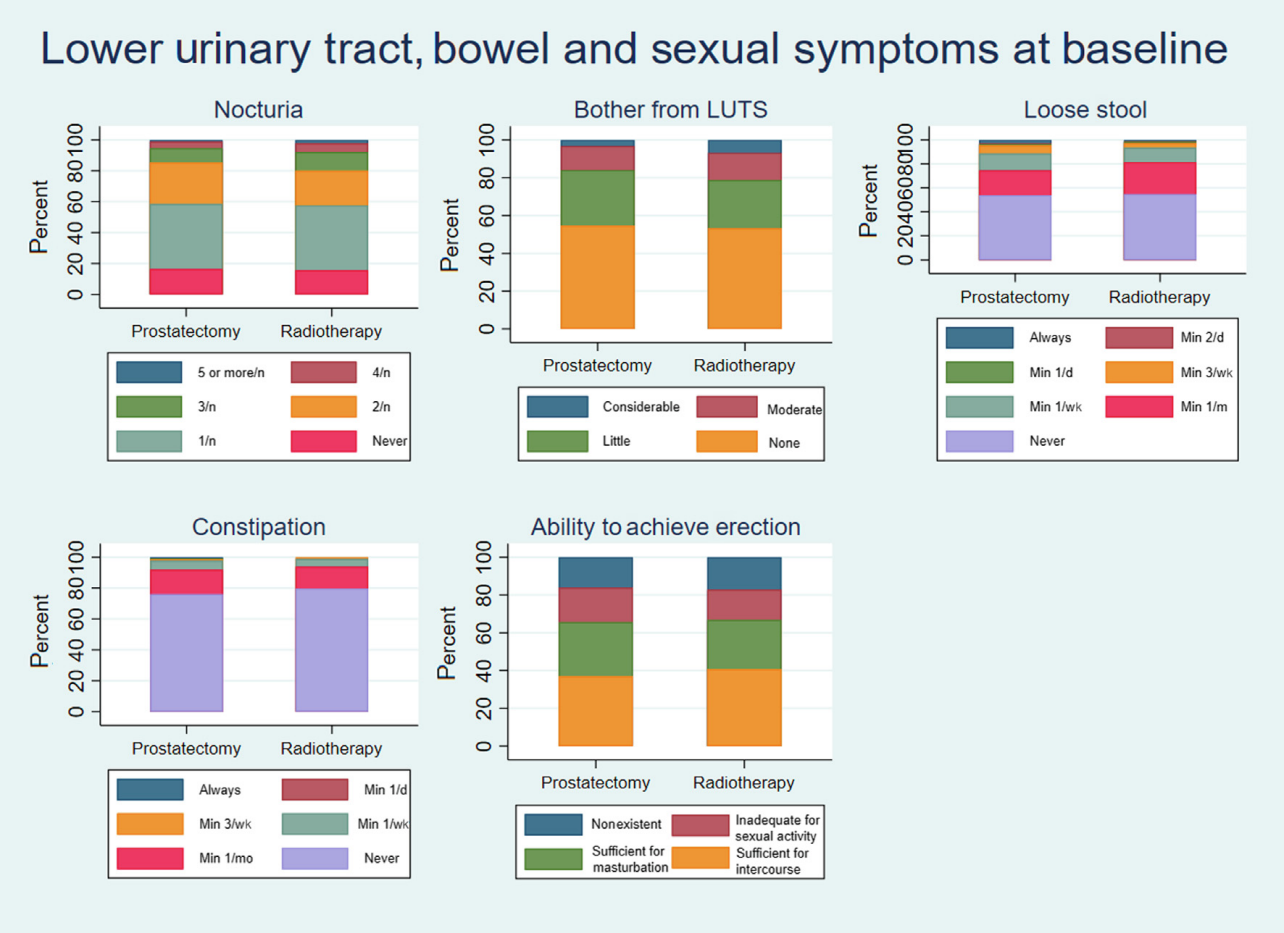
Bar charts showing baseline lower urinary tract, bowel, and sexual symptoms. LUTS = lower urinary tract symptoms; min = minimum; n = night.

At baseline, the men reported low to moderate scores for lower urinary tract symptoms, bowel symptoms, and sexual life (Fig. 1). Of the patients, 3% reported urinary leakage at least once a day, 72% had nocturia up to two times nightly, and 76% stated no to little bother from lower urinary tract symptoms. In total, 74% and 51% of patients reported never experiencing constipation and loose stool ever, respectively. Of the patients, 57% reported having an active sex life with a partner, 62% stated erectile function sufficient for intercourse or masturbation, and 61% reported moderate to very good ability to achieve orgasm.

**Fig. 2:**
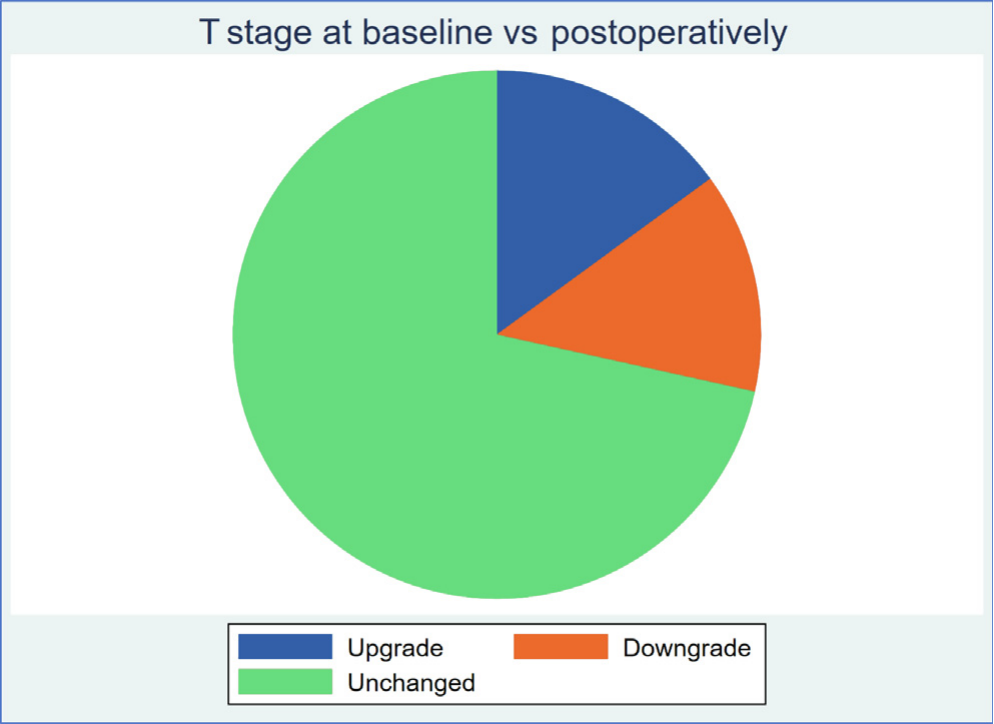
Pie chart showing T stage up- and downgrading rates preoperatively versus postoperatively.

## 4 Discussion

With the randomization of more than 800 men in the SPCG-15 study, we have proved the feasibility of a randomized trial comparing primary surgery with radiotherapy among men with locally advanced prostate cancer. After having randomized the first 600 men, we find balanced distribution of characteristics in the two study arms, and the enrolled men seem to well represent the target population of men with locally advanced cancer (eligible for both treatments) undergoing treatment with curative intent.

Although new treatments are being added to the armory of therapies for prostate cancer, to our knowledge, no new evidence has emerged disputing the need for this trial. The potential benefit of surgery in locally advanced prostate cancer remains unclear, and the rationale for this trial remains. The role of radiotherapy plus androgen deprivation therapy in this patient group is well established [5], [6], [7], and although several observational studies indicate an equal effect of radical prostatectomy—in addition to the SPCG-4 trial showing a clear benefit of radical prostatectomy among patients in whom postoperative upgrading is commonplace—a head-to-head comparison is imperative to provide best possible evidence-based care for this patient group [7], [8], [9], [10], [11], [12], [13], [14], [15], [16], [17], [18], [24].

Our study population so far presents men with high-risk features in terms of histopathology, clinical stage, and PSA (Fig. 2). Of the men in the surgery arm, 80% had no or fewer than four positive lymph nodes in the pathology specimen, and may thus be considered to have potentially curable disease [25]. A significant proportion of men in the surgical arm had pT3b, a disease stage known to be associated with particularly rapid progression to disseminated disease [26]. There is a risk that any difference in outcome between the treatment arms is diluted if a large proportion of men have too advanced disease to be potentially cured by the treatment. Our per-protocol, prespecified, stratified analysis by tumor substage and grade is therefore well justified to assess whether there are different effects of treatment in various subcategories not appearing in the overall analysis.

There are currently no studies directly comparable with SPCG-15, as there are no randomized head-to-head comparisons of radiotherapy and surgery among men with locally advanced prostate cancer. When comparing the characteristics of our study population with previous cohorts from curative trials of the efficacy of surgery, our study includes the most advanced cases. In the SPCG-4 trial, where watchful waiting was compared with radical prostatectomy in the pre-PSA and pre-MRI era, pT3 disease was found in 46.5% of the radical prostatectomy patients as compared with 75% in our study thus far. A comparison of our cohort with the SPCG-4 cohort is relevant because SPCG-4 included men with palpatory T1c or T2 disease at baseline, which turned out to be pT3 in almost half the cases, similar to how we now include patients who show signs of T3 tumors on MRI but might have T1c or T2 according to digital rectal examination. While a majority of radical prostatectomy patients in both SPCG-4 and SPCG-15 had a postoperative Gleason sum of 7/ISUP 2 and 3 (55.2% and 56%, respectively), only 13.4% of SPCG-4 patients had a Gleason sum of 8–10, in contrast to 34% of SPCG-15 patients with ISUP 4 and 5 [24]. SPCG-15 patients seem to be presenting with advanced disease in comparison with the TROG 96.01 trial patients. On the contrary, the PIVOT trial, which also compared surgery with conservative treatment, was conducted in the PSA era, and it randomized mainly men with low-risk disease with Gleason grade 6 pathology who, according to the guidelines of today, should have had active monitoring rather than treatment with curative intent.

With a similar median age of 67–68 yr (depending on treatment arm), in TROG 96.01, the patients had higher median PSA of 14.4–16.4 μg/l, with 33–43% having PSA of >20 μg/l, compared with 12 μg/l and 31%, respectively in our cohort. Notably, TROG 96.01 diagnostic biopsy Gleason scores were remarkably lower; 42–46% of patients in TROG 96.01 were diagnosed with Gleason 2–6 compared with 0% (as a result of the exclusion criteria) in SPCG-15, and 15–20% in TROG were diagnosed with Gleason 8–10 compared with 47% in SPCG-15. However, this is probably attributable to TROG 96.01 including patients with T2b and T2c tumors [7]. We acknowledge that the Gleason system has since been replaced with the ISUP system, making direct comparisons difficult. In summary, SPCG-15 is a cohort with such truly locally advanced disease that has not previously been targeted with such high precision.

The anxiety and depression levels were increased compared with age-matched men in the general population of Sweden and England as well as Denmark [27], [28], [29], possibly due to their recent diagnosis of advanced cancer. Around 25% of men in the Swedish and English populations in similar age spans report having anxiety [27], [28]. In our trial, 48% report moderate to severe anxiety. In our cohort, a significantly higher proportion (10%) than in the general population of Sweden (4%) [28] and England (1%) had severe anxiety [27].

The SPCG-7 trial, comparing radiotherapy plus long-term androgen deprivation therapy with life-long androgen deprivation therapy alone in locally advanced prostate cancer (80%) or cT2 with PSA >20 (20%), investigated a similar target group to the SPCG-15 trial, although SPCG-7 was conducted in the pre-MRI era, and results may not be automatically generalizable to all MRI-defined T3 tumors. The baseline quality–of-life characteristics in SPCG-7 and SPCG-15 trials appear to be similar in many ways. Regarding both lower urinary tract symptoms (including urinary leakage) and bowel symptoms, both cohorts present with very low scores, indicating good urinary function at baseline despite advanced tumors. The majority of SPCG-15 patients reported no-to-little bother due to lower urinary tract symptoms, which is comparable with the average of 2/10 on a bother score (where 10 represents maximum bother) in SPCG-7. The sexual function differs between the two cohorts at baseline; 36% of SPCG-15 men present with erectile function sufficient for intercourse, whereas in SPCG-7, the percentage of men was 47% in the androgen deprivation therapy group and 51% in the radiotherapy plus androgen deprivation therapy group [30].

Regarding education, marital status, and occupation, the SPCG-15 cohort correlates well with the general population for this age group in Sweden according to data from Statistics Sweden, the central bureau for statistics in Sweden [31], [32], [33], [34]. The incidence of overweight (body mass index 25–29) is 47% in our cohort, at the same level as in England and slightly higher than that in the general population in the same age groups both in Sweden and in Denmark (43% and 22%, respectively) [35], [36]. Regarding physical activity, 53% of our patients exercised >3 h/wk as compared with 30% of men aged 65–84 yr in the general population of Sweden [37]. Smoking habits are slightly lower in the general population of Sweden; approximately 8% of men aged 65–84 yr smoke [38] compared with 13% of the men in SPCG-15. However, the general Danish population has a higher smoking prevalence (16.9%) in the age span of 65–74 yr, which probably accounts for the higher numbers in our cohort [39]. In the UK, 9% of men aged 65–74 yr smoke [40].

In summary, our data do not indicate any important deviance from age-equivalent male populations in Sweden, Denmark, and England, which would preclude generalizability at least to other Caucasian male populations [3]. We acknowledge, however, that there may be differences in the tumor biology between different ethnic groups that may somewhat hamper generalization of our data to all men with locally advanced prostate cancer.

It is our opinion that successful and speedy completion of this trial depends on adherence to national and/or European Association of Urology guidelines, and that radiotherapy with neoadjuvant hormonal treatment is the standard treatment of choice outside the trial, whereas surgical treatment is offered only as an experimental treatment within SPCG-15. Although this strategy may be considered controversial since surgery is already widespread in locally advanced prostate cancer, the current evidence basis is undoubtedly stronger for radiotherapy, and a head-to-head comparison is the only way to find out whether primary surgery leads to better outcomes.

We acknowledge that the two treatment arms are heterogeneous in terms of allowing different radiotherapy regimens (eg, external bream radiation plus brachytherapy vs external beam radiation alone) as well as varying surgical approaches (eg, open surgery vs robotic laparoscopic surgery, varying lymph node dissection approaches; Table 6, Table 7, Table 8). This has been a pragmatic approach to make the study realizable since clinical practice differs substantially between centers. The principle of the study is to compare best practices available to the patient. We therefore consider this variability a strength for later generalizability. Nevertheless, due to continuous updates of treatment regimens and care processes, an inherent weakness of the study is the fact that the results may already be outdated when the cohort is mature for final analyses—a destiny to which all studies with long-term follow-up are doomed.

**Table 6:** Primary treatment regimens in the two study arms

Primary treatment regimens	Number of patients (%)
*Primary prostatectomy*	
Total number of patients receiving primary prostatectomy	301 (100)
Surgical approach	
Retropubic prostatectomy	39 (13)
Laparoscopic prostatectomy	3 (1)
Robot-assisted laparoscopic prostatectomy	232 (77)
Missing	27 (9)
Lymph node dissection	
Yes	258 (86)
No	12 (4)
Missing	31 (10)
*Primary radiotherapy*	
Total number of patients receiving primary radiotherapy	299(100)
Radiotherapy regimen	
External beam radiotherapy only	164 (55)
EBRT + high-dose rate brachytherapy	96 (32)
Missing	39 (13)

EBRT = external beam radiotherapy.

**Table 7:** External beam radiotherapy regimens

Total dose prostate (Gy)	Fraction size (Gy)	Number of patients (%)
60–66	3	4 (2)
72.5–77	2.3–2.7	7 (4)
77	2.2	12 (7)
74–78	1.8–2	141 (86)
All	All	164 (100)

**Table 8:** External beam radiotherapy plus high-dose rate brachytherapy regimens

Total dose, prostate (Gy)	External beam radiotherapy total dose/dose fraction (Gy)	Brachytherapy dose (Gy) × number of fractions	Number of patients (%)
70	50/2	10 × 2	50 (52)
65	50/2	15 × 1	40 (42)
70	60/2	10 × 1	1 (1)
60	50/2	10 × 1	1 (1)
55	40/2	15 × 1	1 (1)
Missing	Missing	15 × 1	1 (1)
Missing	Missing	10 × 2	2 (2)
All	All	All	96 (100)

## 5 Conclusions

**Fig. 3:**
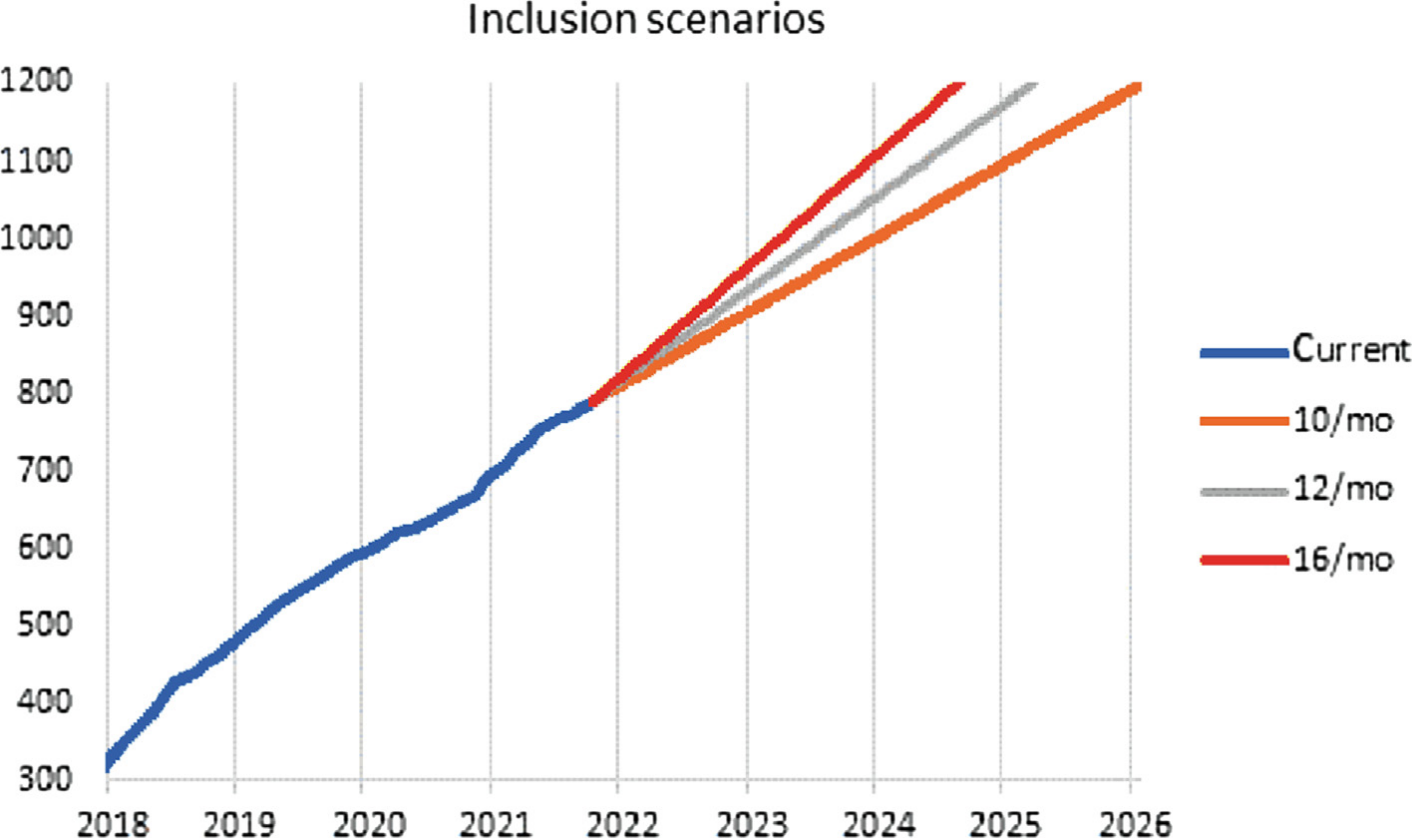
Current and past inclusion rates as well as prediction of future inclusion rates.

In summary, we are presenting a cohort with advanced prostate cancer disease with respect to both stage and grade. Altogether, our study population seems to well represent the Scandinavian population at large. SPCG-15 has demonstrated that randomization to surgery or radiotherapy is feasible in a population of men with locally advanced prostate cancer. We are two-thirds through its inclusion phase and are likely to be closed for further inclusion within 4 yr (Fig. 3).

  ***Author contributions*:** Magdalena Gongora had full access to all the data in the study and takes responsibility for the integrity of the data and the accuracy of the data analysis.

*Study concept and design*: Gongora, Akre, Brasso, Petersen, Lilleby, Mirtti, Jäderling, Bottai, Stranne, Karlsson, Johansson, Vincent.

*Acquisition of data*: Brasso, Stranne, Akre, Petersen, Lilleby, Mirtti, Jäderling, Bottai, Karlsson, Johansson, Jacobsen, Hansen, Rannikko, Olsson, Lindberg, Røder.

*Analysis and interpretation of data*: Gongora, Stranne, Brasso, Petersen, Lilleby, Mirtti, Jäderling, Bottai, Karlsson, Johansson, Vincent, Jacobsen, Hansen, Rannikko, Olsson, Lindberg, Røder.

*Drafting of the manuscript*: Gongora, Akre, Stranne, Johansson, Vincent.

*Critical revision of the manuscript for important intellectual content*: Gongora, Stranne, Akre, Brasso, Petersen, Lilleby, Mirtti, Jäderling, Bottai, Karlsson, Johansson, Vincent, Jacobsen, Hansen, Rannikko, Olsson, Lindberg .Røder.

*Statistical analysis*: Gongora, Bottai.

*Obtaining funding*: Akre.

*Administrative, technical, or material support*: Vincent.

*Supervision*: Akre, Stranne.

*Other*: None.

  ***Financial disclosures:*** Magdalena Gongora certifies that all conflicts of interest, including specific financial interests and relationships and affiliations relevant to the subject matter or materials discussed in the manuscript (eg, employment/affiliation, grants or funding, consultancies, honoraria, stock ownership or options, expert testimony, royalties, or patents filed, received, or pending), are the following: Magdalena Gongora has received a research scholarship connected to her position both as a PhD student and as a urology resident by Forskningsrådet KI-Region Stockholm (Research Council KI-Region Stockholm; grant number FoUI-955436). Antti Rannikko is a member of the board in Ida Montin Foundation and Orion Research Foundation; advisory board member for medical companies Bayer, Orion Pharma, and Janssen; and clinical advisor for Aqsens, company for which he has stock and investigator in clinical trials by Rho-Vac, Orion Pharma, Bayer, Astellas, Pfizer, and Janssen. Henrik Jakobsen has honoraria for education of colleagues sponsored by Bayer, Astellas, and MSD (2022). Fredrik Jäderling has financing from the Swedish state under the agreement between the Swedish government and the county councils (the ALF-agreement) 2021–2023, and holds presentation funded by AstraZeneca. Tuomas Mirtti has financing from Academy of Finland (grant no. 323098) and Cancer Foundation Finland (no specific grant number).

  ***Funding/Support and role of the sponsor*:** Study sponsor is Region Stockholm represented by Karolinska University Hospital. The study was financed by grants from the Swedish Research Council (dnr 2017-00546), the Nordic Cancer Union, and the Swedish state under the agreement between the Swedish government and the county councils (the ALF-agreement; grant number FoUI-953889). Individual author grants have been declared at submission. This work was supported in part by a grant from the Finnish Cancer Organisations (to Assistant Professor Rannikko) and Jane and Aatos Erkko Foundation.
